# Derivation of first-order dissolution rates to estimate particle clearance and burden in the human respiratory tract

**DOI:** 10.1186/s12989-023-00523-z

**Published:** 2023-04-27

**Authors:** James S. Brown, Gary L. Diamond

**Affiliations:** 1 U.S. Environmental Protection Agency, Office of Research and Development, 109 TW Alexander Drive, Mail Code B243-01, Research Triangle Park, NC 27711 USA; 2 SRC, Inc., North Syracuse, NY USA

## Abstract

Inhalation is a portal-of-entry for aerosols via the respiratory tract where particulate burden accumulates depending on sites of particle deposition, normal clearance mechanisms, and particle solubility. The time available for dissolution of particles is determined by the balance between the rate of particle clearance from a region and their solubility in respiratory solvents. Dissolution is a function of particle surface area divided by particle volume or mass (i.e., dissolution is inversely proportional to the physical diameter of particles). As a conservative approach, investigators commonly assume the complete and instantaneous dissolution of metals from particles depositing in the alveolar region of the respiratory tract. We derived first-order dissolution rate constants to facilitate biokinetic modeling of particle clearance, dissolution, and absorption into the blood. We then modeled pulmonary burden and total dissolution of particles over time as a function of particle size, density, and solubility. We show that assuming poorly soluble particle forms will enter the blood as quickly as highly soluble forms causes an overestimation of concentrations of the compound of interest in blood and other extrapulmonary tissues while also underestimating its pulmonary burden. We conclude that, in addition to modeling dose rates for particle deposition into the lung, physiologically based pharmacokinetic modeling of pulmonary and extrapulmonary tissues concentrations of moderately and poorly soluble materials can be improved by including estimates of lung burden and particle dissolution over time.

## Introduction

Particle dissolution in the respiratory tract depends on particle size, solubility, site of deposition, and subsequent disposition of the particles (e.g., mucociliary clearance to the gastrointestinal [GI] tract, macrophage phagocytosis, translocation to lymph, etc.). Dissolution is the dissociation of material from particles in the lung into a form that may be absorbed into the blood. Physiologically based pharmacokinetic (PBPK) models that simulate distribution, metabolism and excretion of inhaled particles must also simulate rates of particle deposition, dissolution and absorption within the respiratory tract.

The International Commission on Radiological Protection (ICRP) provides recommendations and guidance on protection against the risks associated with ionizing radiation from natural sources, medical sources, general industry, and nuclear enterprises. In several publications, the ICRP has recommended categorizing inhaled particles into classes that represent fast (F), moderate (M), or slow (S) rates of dissolution. Each class is assigned distinct values for parameters that govern particle dissolution and absorption into blood. In the absence of information suggesting otherwise, ¶58 of Ref. [1] recommends that most elements be classified as Type M since it is the least likely to substantially over- or under-estimate dissolution and absorption. However, a conservative approach in applications of PBPK to risk assessment of elements that produce systemic effects (i.e., outside of the respiratory tract), is to assume the complete and instantaneous dissolution metals from particles depositing in the alveolar region of the respiratory tract, e.g., see Ref. [2] for manganese (Mn) and Ref. [3, 4] for lead (Pb).

While appearing logically sound as a conservative risk assessment assumption, we will show in this analysis that, for particles having slow rates or moderate rates of dissolution, assuming rapid dissolution greatly overestimates the amount of material available for absorption into the blood. For poorly soluble particles (i.e., Type S dissolution), depending on particle size, it may take decades to achieve a steady state alveolar retention in which a balance is reached between rates of particle deposition, particle clearance (transport of particles between respiratory compartments), and dissolution. The maximal rates of poorly soluble particle dissolution will not be achieved until steady state alveolar and interstitial retention is reached. However, even at steady state, the rates of poorly soluble particle dissolution will never achieve those of highly soluble (i.e., Type F dissolution) particles because much of the poorly soluble particle mass will have been cleared by pulmonary clearance to the tracheobronchial airways from which they are then cleared to the gastrointestinal tract. Thus, assuming overly fast dissolution and absorption of poorly soluble particles deposited in the lungs causes an overestimation the distribution and burden in extrapulmonary tissues while also underestimating pulmonary burden.

We agree with Ref. [5] that it is too simplistic to group particles into just soluble and poorly soluble materials. In their review of nanoparticles, the authors note that dissolution rates are much faster for smaller than for larger particles. Additionally, they conclude that effective clearance rates are a function of both particle clearance and dissolution. Conceptually, the authors suggest that using differences in the pulmonary clearance/retention kinetics between inhaled poorly soluble and soluble particles can be employed to determine the in vivo dissolution rates. The authors validated this concept for determining in vivo dissolution with reference to results of published studies. In our analysis, we have relied on studies [5–7], and several ICRP reports, as a basis for a model of particle dissolution in the respiratory tract that can be applied in PBPK models of inhaled particulates.

In consideration of particle dissolution and absorption into the blood, we made several basic assumptions based on ICRP recommendations that are more fully described in the Methods section. Based on Ref. [8] Annex E and Ref. [9] Annex A, we assumed that the time available for dissolution of particles is determined by the balance between the rate of particle clearance from a respiratory tract region and the rate of particle dissolution. Consistent with ¶E36 of Ref. [8], we assume that the rate of particle dissolution depends on both a dissolution rate constant and surface area of particles deposited in the respiratory tract. Consistent with ¶E48 of Ref. [8], we also assumed normal particle clearance is independent of material type (e.g., species of metal) and that particle clearance is independent from particle dissolution and absorption into the blood. Consistent with ¶101 of Ref. [9], we assume that absorption into blood depends on the physical and chemical form of the deposited material. In contrast to ¶105 of Ref. [9] which assumes that dissolution rates decrease with time, but consistent with ¶E36 of Ref. [8], we assumed the particle dissolution rates increase over time since as particles dissolve, they become smaller and their surface area per mass increases. Ultimately, we derive first-order dissolution rate constants to facilitate biokinetic modeling of particle clearance, dissolution, and absorption.

## Methods

### Dissolution rates

We assume that dissolution is a function of particle surface area divided by particle volume or mass, i.e., dissolution is inversely proportional to the equivalent volume diameter of particles. An equivalent volume diameter is the physical diameter of a sphere having the same volume and density as the irregular shaped particle being considered (see ¶D23–26 of Ref. [8]). As theoretical rates of particle dissolution can be presented independently of particle deposition in and clearance from the respiratory tract, we derive equations for dissolution first. The approximate fraction dissolved per day (*f*_dr,initial_) of particles having an initial median physical particle size d_r,initial_ in cm deposited in a region *r* of the respiratory tract is estimated by Eq. 1 based on Ref. [7] and Section E.2.2.1 of Ref. [8].

1 $$f_{{d_{r,initial} }} = k\left( {{{\pi d_{r,initial}^{2} } \mathord{\left/ {\vphantom {{\pi d_{r,initial}^{2} } {\frac{{\pi \rho d_{r,initial}^{3} }}{6}}}} \right. \kern-0pt} {\frac{{\pi \rho d_{r,initial}^{3} }}{6}}}} \right) = {{6k} \mathord{\left/ {\vphantom {{6k} {\rho d_{r,initial} }}} \right. \kern-0pt} {\rho d_{r,initial} }}$$

where *k* is the dissolution rate (g/cm^2^ particle surface area per day), and *ρ* is particle density (g/cm^3^). Note that *ρ* is particle density itself and is not adjusted for the amount of the material of concern (e.g., Mn or Pb) in a particle, which affects its density. Particle dissolution rates increase over time since, as particles dissolve, they become smaller and their surface area per mass increases.

Expressing the relationship in Eq. 1 as a different equation for particle mass remaining gives Eq. 2.

2 $$\frac{{{\text{dm}}}}{dt} = \frac{d}{dt}\frac{\pi }{6}\rho d_{r,initial}^{3} = - k\pi d_{r,initial}^{2}$$

Rearranging and expressing a differential equation for particle diameter gives Eq. 3.

3 $$\frac{d}{dt}\frac{\pi }{6}\rho d_{r,initial}^{3} = \frac{\pi }{2}\rho d_{r,initial}^{2} \frac{{dd_{r,initial} }}{dt} = - k\pi d_{r,initial}^{2}$$

The solutions for instantaneous particle size, remaining particle mass, and dissolution rate over time in each lung region are given by Eqs. 4, 5, and 6, respectively.

4 $$d_{r} \left( t \right) = d_{r,initial} - \frac{2k}{\rho }t$$

5 $$m\left( t \right) = \frac{\pi }{6}\rho \left( {d_{r,initial} - \frac{2k}{\rho }t} \right)^{3}$$

6 $$dissolution\left( t \right) = k\pi \left( {d_{r,initial} - \frac{2k}{\rho }t} \right)^{2}$$

Although it is possible to solve for instantaneous particle parameters in Eqs. 4–6, we approximate this relationship using first-order dissolution rate constants for several reasons. First, the use of first-order dissolution rates is consistent with the use of first-order mass transfer rates among lung compartments in models of lung clearance, see Ref. [8] Annex E and Ref. [9] Annex A. Second, as reviewed in Section 4.3.1.3 and of Ref. [10], macrophage related alveolar particle clearance and particle movement across the epithelium into interstitial space and lymphatics are dependent on particle size. Third, as discussed in Section 4.3.1.2 of Ref. [10], clearance kinetics of the tracheobronchial region are based on experimental studies that lack resolution with regard to the anatomical site of particle deposition and particle size related effects on clearance are a subject of debate. Fourth, it is unknown how particle clearance mechanisms would affect the time-dependent size distribution of particles available for dissolution over time. Accordingly, we derive first-order dissolution rates as described below.

The time (t_*frac*_) for a particle to reach a specific fraction of mass remaining (m_*frac*_) of greater than zero is given by Eq. 7.

7 $$t_{frac} = \frac{\rho }{2k}d_{r,initial} \left( {1 - \frac{1}{{m_{frac}^{ - 1/3} }}} \right)$$

Assuming that a particle is not cleared from a lung region, the time to reach zero particle mass remaining by dissolution is obtained from Eq. 7 by setting the parenthetical to one. Note that within Eqs. 1–7, particle diameter must be converted from µm to cm for consistency with units of *k* and *ρ*.

First-order particle dissolution rates (λ_r_) for particles were calculated for fractions of mass remaining from 0.01 to 0.99 by Eq. 8.

8 $$\lambda_{r} = {{ - ln\left( {m_{frac} } \right)} \mathord{\left/ {\vphantom {{ - ln\left( {m_{frac} } \right)} {t_{frac} }}} \right. \kern-0pt} {t_{frac} }}$$

Goodness of fit (*r*^2^) for first-order dissolution rates estimated by Eq. 8 was then calculated relative to the particle mass remaining over time from Eq. 5. The *r*^2^ were calculated as the model sum of squares (MSS) divided by the total corrected sum of squares (TSS). The MSS equals the TSS minus the residual sum of squares (RSS). In typical linear regressions, when a model is fitted to a data set, the resulting *r*^2^ must be nonnegative since the least square fitting procedure assures RSS ≤ TSS. When *r*^2^ is computed on excluded data, that is, data not used to fit the model, RSS can exceed the TSS. In this case, *r*^2^ (which is not the squared number) can be negative, indicating that the mean of the data is a better predictor than the model.

### Particle dose rates

The efficiency of particle deposition in the respiratory tract depends, in large part, on inhaled particle size and shape, route of breathing (nasal or oronasal), tidal volume (V_T_), breathing frequency (f), and respiratory tract morphology. The route of breathing (i.e., the distinction between air passing through the nose vs. the mouth) is important because the nasal passages more effectively remove inhaled particles than the oral passage. For a detailed description of these factors, readers are referred to the review provided in Section 4.1 of Ref. [10]. Section 4.2 of that document reviews particle deposition in the respiratory tract and factors modulating deposition.

We used the publicly available multiple-path particle dosimetry (MPPD) model version 3.04 to estimate particle deposition efficiencies for 301 monodisperse particles equally spaced on a log scale of particle diameter from 0.001 and 100 µm for varied particle densities (0.5–12 g/cm^3^) and human activity levels [11]. The MPPD model can be used to calculate particle deposition and clearance in multiple species. A description of the model, recent model improvements, and advancements incorporated into the MPPD model are provided elsewhere [12]. For brevity, only the results of specific conditions will be presented here. Respiratory parameters used in the MPPD model are provided in Table 1. All simulations used an “Oronasal-Mouth Breather” breathing scenario (also termed as Breathing Habit). This breathing scenario was chosen since it is more conservative (greater particle deposition in lower respiratory tract, i.e., TB and PU regions) than nasal or oronasal-normal augmenter breathing scenarios. The symmetric lung morphology was used for all simulations [13].

**Table 1:** Respiratory parameters for male and female adults

Sex	Respiratory parameter	Sitting	Light exercise	Heavy exercise
Male	V_T_ (L)^a^	0.750	1.250	1.923
	f (min^−1^)^a^	12	20	26
	Breathing route^b^	0.3, 0.7	0.6, 0.4	0.7, 0.3
	FRC (L)^a^	3.30		
	URT (mL)^a^	50		
Female	V_T_ (L)^a^	0.464	0.992	1.364
	f (min^−1^)^a^	14	21	33
	Breathing route^b^	0.3, 0.7	0.6, 0.4	0.7, 0.3
	FRC (L)^a^	2.68		
	URT (mL)^a^	40		

*f* breathing frequency, *FRC* functional residual capacity (assumed constant among activities), *URT* upper respiratory tract volume (assumed constant among activities), *V*_*T*_ tidal volume

^a^Data from Table B.15 of Ref. [8]

^b^Values are the fraction of breath entering through the mouth and nose (fraction mouth, fraction nose) used in MPPD model version 3.04 for oronasal-mouth breather

With basic lognormal aerosol parameters (mass median particle size, d_50%_; and the geometric standard deviation, σ_g_ also GSD), the particle size (d_i_) associated with any percentile (P) of the distribution can be calculated by Eq. 9.

9 $$d_{i} = d_{50\% } \sigma_{g}^{z\left( P \right)}$$

where z(P) is the number of standard deviations from the mean for the normal distribution at a given cumulative probability P provided by a standard normal table or Z-table. We used Eq. 9 to estimate the particle size associated with each fraction (namely 0.005) of particle size distribution(s) of interest from the 0.005 to the 0.995 percentile of the aerosol distribution. The deposition fraction (DF_r,di_) of each particle size d_i_ in the aerosol distribution in region r of the respiratory tract was calculated by linear interpolation between the 301 monodisperse particles in MPPD output. Since the mass was evenly divided into 0.50% increments, the concentration (µg/m^3^) of the compound of interest associated with each particle size interval (Cd_i_) is the airborne concentration of that compound (µg/m^3^) times 0.005.

The mass deposition rate ($\dot{M}_{{r,d_{i} }}$) in µg/day in each respiratory region r (i.e., ET, TB, PU) is given by Eq. 10.

10 $$\dot{M}_{{r,d_{i} }} = DF_{{r,d_{i} }} C_{{d_{i} }} V_{T} \left( {\frac{{{\text{m}}^{3} }}{{1000 \,{\text{L}}}}} \right)f\left( {\frac{60\,\min }{{\text{hr}}}} \right)t$$

where DF_r,di_ is the deposition fraction of particle size d_i_ in region r of the respiratory tract, Cd_i_ is the inhaled concentration (µg/m^3^) of the compound of interest associated with each particle size interval, V_T_ is tidal volume (liters), *f* is breathing frequency (min^−1^), *t* is number of hours per day that a person is exposed to the aerosol at a specific activity level which determines V_T_ and *f*, and the parentheticals are unit conversion factors. The overall mass deposition rate ($\dot{M}_{r}$) in each respiratory tract region is the sum of deposition rates across all particle sizes $\dot{M}_{{r,d_{i} }}$ for that region (Eq. 11).

11 $$\dot{M}_{r} = \mathop \sum \limits_{i = 1}^{n} \dot{M}_{{r,d_{i} }}$$

Although not used herein, the overall deposition fraction (DF_r_) in each respiratory tract region is given by Eq. 12.

12 $$DF_{r} = {{\dot{M}_{r} } \mathord{\left/ {\vphantom {{\dot{M}_{r} } {C V_{T} \left( {\frac{{{\text{m}}^{3} }}{{1000\,{\text{L}}}}} \right)f\left( {\frac{60\,\min }{{{\text{hr}}}}} \right)}}} \right. \kern-0pt} {C V_{T} \left( {\frac{{{\text{m}}^{3} }}{{1000\,{\text{L}}}}} \right)f\left( {\frac{60\,\min }{{{\text{hr}}}}} \right)}}$$

where C is the inhaled concentration (µg/m^3^) of the compound of interest.

For particle burden and dissolution (subsequent section) calculations, it was necessary to estimate the initial median particle size (d_r,initial_) depositing in each respiratory tract region. The d_r,initial_ was estimated as the d_i_ corresponding to a z-value of zero for the cumulative fraction of $\dot{M}_{{r,d_{i} }}$ of $\dot{M}_{r}$ in Eq. 11.

### Particle burden and dissolution

**Fig. 1:**
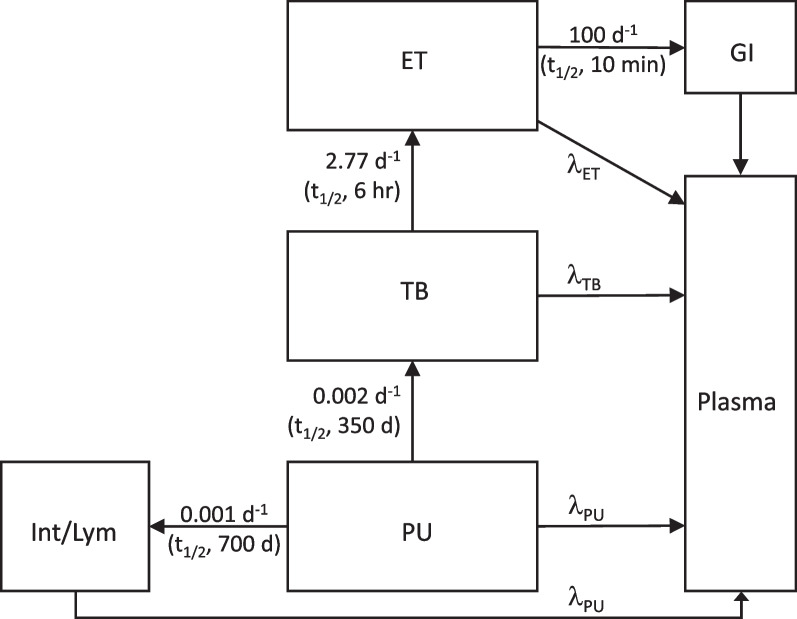
Compartmental model used to represent mass transfer among regions. ET, extrathoracic; GI, gastrointestinal; Int/Lym, interstitial and lymphatic; PU, pulmonary; TB, tracheobronchial; and λ_ET_, λ_TB_, and λ_PU_ are first-order rate constants for dissolution for respiratory tract regions

Figure 1 illustrates our compartmental model to estimate first-order mass transfer rates of compounds of interest (e.g., Mn and Pb) that may be associated with particles deposited in the respiratory tract. This figure is based on Ref. [8] Annex E and Ref. [9] Annex A. Consistent with ¶E48 of Ref. [8], we assumed normal particle clearance (transport of particles themselves among respiratory compartments) is independent of material type and that particle clearance is independent from particle dissolution and absorption into the blood. Accordingly, each respiratory compartment (e.g., the pulmonary compartment, PU) has first-order rates of clearance to other compartments (indicated by a rate in d^−1^ and half-time) as well as a first-order rate constant for dissolution (indicated by a λ_r_ for each region).

For simplicity relative to Ref. [9], we have combined the interstitial and lymphatic compartments since their only path for removal is dissolution, which will be the same as from the pulmonary region. With consideration to the literature reviewed in Section 4.3.1.2 of Ref. [10] describing clearance from tracheobronchial region, we grouped the bronchioles and bronchi into a single tracheobronchial (TB) compartment in our model. We applied a single rate constant equivalent to a half-time of 6 h because mucociliary clearance from the TB region is generally considered to be a rapid process that is relatively complete by 24–48 h post-inhalation in healthy humans. Our model does not include sequestration compartments for particles deposited in the ciliated airways. Our rationale for excluding particle sequestration was a concern that existence of an apparent slow ciliated airway clearance and sequestration may be due to experimental artifacts. For example, Ref. [14] showed particle deposition in the alveolar region even with shallow inhalations and Ref. [15] showed that ventilation distribution affects patters of particle deposition in health and disease. Thus, the experimental artifact causing apparent slow ciliated airway clearance is due to nonuniform ventilation distribution with some inhaled particles reaching and depositing within anatomically deeper lung regions (beyond the ciliated airways) than expected under the assumption of uniform and symmetric filling of lung regions.

Pulmonary burden (BPU) at time *t* resulting from deposited particle mass deposited may be estimated using Eq. 13.

13 $$B_{PU} \left( t \right) = \dot{M}_{PU} \Delta t + B_{PU} \left( {t - \Delta t} \right)e^{{ - \left( {\lambda_{PU,Clr} + \lambda_{Int} + \lambda_{{{\text{PU}}}} } \right)\Delta t}}$$

where $\dot{M}_{PU}$ is the mass deposition rate (see Eq. 8) in the pulmonary region of a compound in µg/day, λ_PU,Clr_ is the first-order rate constant (0.002 d^−1^; t_1/2_, 350 days) for normal pulmonary (PU) clearance into the bronchioles; λ_PU_ is the first-order rate constant for dissolution of particles deposited in the PU region; λ_Int_ is the first-order rate constant (0.001 d^−1^; t_1/2_, 700 days) for transfer from the PU region into the interstitial space and lymphatics, and ∆t is the time-step. The majority of particle burden in the respiratory tract is in the PU region. Accordingly, the majority of particle dissolution occurs in the PU region.

The dissolution (Dissol_PU_) or dissociation of the compound of interest from particles deposited in the PU region is then estimated in Eq. 14.

14 $$\begin{aligned}Dissol_{PU} \left( t \right) &= \frac{{\lambda_{PU} }}{{\lambda_{PU,Clr} + \lambda_{PU} + \lambda_{Int} }}B_{PU} \left( {t - \Delta t} \right)\\ & \quad \left[ {1 - e^{{ - \left( {\lambda_{PU,Clr} + \lambda_{Int} + \lambda_{PU} } \right)\Delta t}} } \right]\end{aligned}$$

Interstitial particle burden (BInt) and dissolution (Dissol_Int_) at time *t* is estimated using Eq. 15 and 16, respectively.

15 $$\begin{aligned} B_{Int} \left( t \right) &= B_{Int} \left( {{\text{t}} - \Delta t} \right) + \frac{{\lambda_{Int} }}{{\lambda_{PU,Clr} + \lambda_{PU} + \lambda_{Int} }}B_{PU} \left( {t - \Delta t} \right)\\ & \quad \left[ {1 - e^{{ - \left( {\lambda_{PU,Clr} + \lambda_{Int} + \lambda_{{{\text{PU}}}} } \right)\Delta t}} } \right] \end{aligned}$$

16 $$Dissol_{Int} \left( t \right) = B_{Int} \left( {{\text{t}} - \Delta t} \right)\left[ {1 - e^{{ - \lambda_{PU} \Delta t}} }\right]$$

Herein, we have not modeled the absorption of dissolved particle constituents into blood and assume that all dissolved material is available for absorption (i.e., absorption fraction of dissolved material is 100%). We recognize that, depending on the compound of interest, it may become bound to proteins or other substances and that such reactions could alter the rate or extent of absorption into the blood.

For relatively soluble particles, burden and dissolution may also be important for particles deposited in the TB and ET regions. Tracheobronchial burden (B_TB_) results from both deposition in this region and clearance from the PU region to the TB region as indicated by Eq. 17.

17 $$\begin{aligned} B_{TB} \left( t \right) &= \dot{M}_{TB} \Delta t + B_{TB} \left( {t - \Delta t} \right)e^{{ - \left( {\lambda_{TB,Clr} + \lambda_{TB} } \right)\Delta t}} \\ & \quad + \frac{{\lambda_{PU,Clr} }}{{\lambda_{PU,Clr} + \lambda_{PU} + \lambda_{Int} }}B_{PU} \left( {t - \Delta t} \right)\\ & \quad \left[ {1 - e^{{ - \left( {\lambda_{PU,Clr} + \lambda_{{{\text{PU}}}} + \lambda_{Int} } \right)\Delta t}} } \right]\end{aligned}$$

where λ_TB,Clr_ is a first-order rate constant of 2.77 d^−1^ (half-time, 6 h) for normal mucociliary clearance from the tracheobronchial airways. Likewise, extrathoracic burden (B_ET_) results from both particles depositing in this region and those cleared from the TB region as indicated by Eq. 18.

18 $$\begin{aligned} B_{ET} \left( t \right) &= {\dot{M}}_{ET} \Delta t + B_{ET} \left( {t - \Delta t} \right)e^{{ - \left( {\lambda_{ET,Clr} + \lambda_{ET} } \right)\Delta t}} \\& \quad + \frac{{\lambda_{TB,Clr} }}{{\lambda_{TB,Clr} + \lambda_{TB} }}B_{TB} \left( {t - \Delta t} \right)\\ & \quad \left[ {1 - e^{{ - \left( {\lambda_{TB,Clr} + \lambda_{TB} } \right)\Delta t}} } \right] \end{aligned}$$

where λ_ET,Clr_ is a first-order rate constant of 100 d^−1^ (half-time, 10 min) for normal mucociliary clearance from the extrathoracic region.

The dissolution (Dissol_r_) of material from particles deposited in the ET and TB regions is estimated using Eq. 19.

19 $$Dissol_{r} \left( t \right) = { }\frac{{\lambda_{r} }}{{\lambda_{r,Clr} + \lambda_{r} }}B_{r} \left( {t - \Delta t} \right)\left[ {1 - e^{{ - \left( {\lambda_{r,Clr} + \lambda_{r} } \right)\Delta t}} } \right]$$

where the subscript *r* refers to the respiratory tract region (i.e., ET or TB) being solved for.

Similarly, clearance particles from the ET region to the stomach is estimated using  Eq. 20.

20 $$Stomach_{ET} \left( t \right) = { }\frac{{\lambda_{ET,Clr} }}{{\lambda_{ET,Clr} + \lambda_{ET} }}B_{ET} \left( {t - \Delta t} \right)\left[ {1 - e^{{ - \left( {\lambda_{ET,Clr} + \lambda_{ET} } \right)\Delta t}} } \right]$$

For gastrointestinal tract absorption in adults, we assumed 9% absorption for metals mimicking calcium such as lead. Gastrointestinal absorption of Pb from soil in fasted (2.5%) and fed (26%) adults has been reported [16]. An absorption of 9% for adults is equivalent to concluding that material cleared from the respiratory tract over the course of a 24-h day will reach a fed stomach about 1/3 of the time. For the essential metal, Mn, Ref. [17] recommends 5% absorption for Type F (Fast; e.g., MnSO_4_), 1% for Type M (Moderate), and 0.05% for Type S (Slow; e.g., MnO_2_) forms of Mn.

## Results

### Dissolution rates

**Fig. 2:**
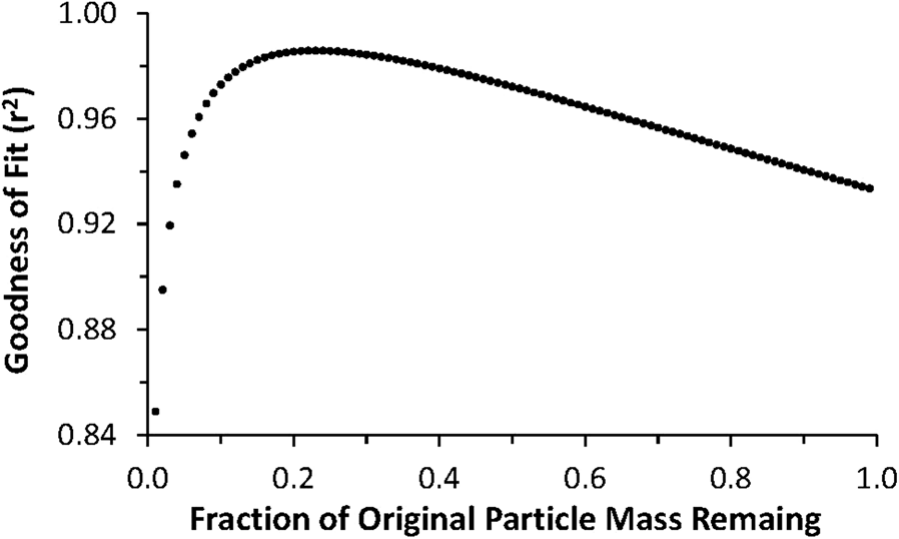
Optimization of fit for dissolution first-order rate constant. Circles are the goodness of fit of first-order dissolution rates (λ_r_) calculated based on the time to reach specified fractions of initial particle mass remaining

**Fig. 3:**
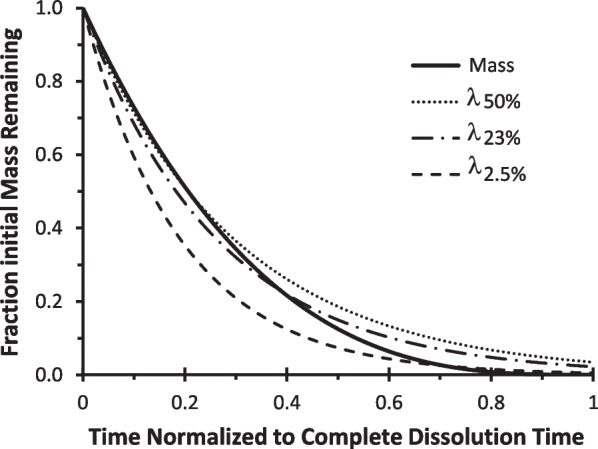
Remaining particle mass (solid line) over time for an initial particle size of 0.5 µm with ρ = 3.0 g/cm^3^ and k = 1 × 10^−6^ g/cm^2^ per day (moderately soluble). The dotted and dashed lines (λ_%_) are the remaining mass approximated using first-order rate constants estimated based on the time to reach specified percentages of mass remaining

Dissolution rate changes with decreasing particle size which results in a nonlinear decrease in particle mass with time. This nonlinear decrease in particle mass was approximated with first order dissolution rates calculated for fractions of mass remaining from 0.01 to 0.99. Figure 2 illustrates the goodness of fit for first-order dissolution rates estimating using Eq. 8. The goodness of fit was maximized by estimating the first-order dissolution rate at 23% of initial particle mass remaining. Figure 3 illustrates particle mass remaining over time (Eq. 5) normalized by the time required for full particle dissolution and predicted mass remaining based on first-order dissolution rates estimated based on the time to reach 50%, 23%, and 2.5% of the initial mass remaining. While the first-order dissolution rate at 50% mass remaining well captures early rates of dissolution, it badly underestimated dissolution at later time points. Conversely, the estimated first-order dissolution rate at 2.5% mass remaining well captures dissolution at later time points, but greatly overestimated dissolution occurring at earlier time points. The first-order dissolution rate at 2.5% mass remaining was nearly identical to an exponential decay fitted with a fixed intercept of 1.0 to the mass remaining curve versus time. Based on Figs. 2 and 3, the first-order dissolution rate based on the time to 23% remaining mass best approximate overall dissolution rates as particle size changed over time. Optimization at 23% mass remaining is invariant to particle size, density, and dissolution rate.

Table 2 shows the time to reach 23% of d_r,initial_ mass remaining as function of initially deposited particle size and dissolution rate. A highly soluble 10 µm particle with a dissolution rate of 1 × 10^−4^ g/cm^2^ per day dissolves to only 23% initial mass remaining in the same amount of time as a moderately soluble 0.1 µm particle with a dissolution rate of 1 × 10^−6^ g/cm^2^ per day. Thus, it is clearly important to consider the particle size depositing in regions of the respiratory tract, especially the alveolar region where retention periods are the longest. For comparison to the ICRP classifications of fast (F), moderate (M), and slow (S) particle types, dissolution rates (*k* in Eq. 1) between 1 × 10^−3^ and 1 × 10^−4^ g/cm^2^ per day are highly soluble (F, dissolution of 1 µm particles in hours), between 1 × 10^−5^ and 1 × 10^−6^ g/cm^2^ per day are moderately soluble (M, dissolution of 1 µm particles in days to months), and between 1 × 10^−7^ and 1 × 10^−8^ g/cm^2^ per day are poorly soluble (S, dissolution 1 µm particles in years to decades), respectively.

**Table 2:** Particle dissolution as a function of dissolution rate and particle size

Dissolution rate, *k* (g/cm^2^ per day)	Initial deposited mass median particle diameter (µm)^a^				
	0.1	0.5	1	5	10
	Time to 23% initial particle mass remaining, days (First-order dissolution rate, d^−1^)				
1 × 10^−3^	0.0058 (253)	0.0290 (50.6)	0.058 (25.3)	0.290 (5.06)	0.581 (2.53)
1 × 10^−4^	0.058 (25.3)	0.290 (5.06)	0.581 (2.53)	2.90 (0.506)	5.81 (0.253)
1 × 10^−5^	0.58 (2.53)	2.90 (0.506)	5.81 (0.253)	29.0 (0.051)	58.1 (0.0253)
1 × 10^−6^	5.81 (0.253)	29.0 (0.051)	58.1 (0.0253)	290 (0.0051)	581 (0.0025)
1 × 10^−7^	58.1 (0.0253)	290 (0.0051)	581 (0.0025)	2905 (0.0005)	5810 (0.0003)
1 × 10^−8^	581 (0.0025)	2905 (0.0005)	5810 (0.0003)	29,050 (0.00005)	58,100 (0.00003)

^a^Dissolution calculations are for a particle density of 3 g/cm^3^ in this example. If density was doubled to 6 g/cm^3^, the time to 50% dissolution would also be doubled and the first-order rate constant would be halved. Conversely, if density was halved to 1.5 g/cm^3^, the time to 50% dissolution would also be halved and the first-order rate constant would be doubled

### Particle dose, burden, and dissolution

**Fig. 4:**
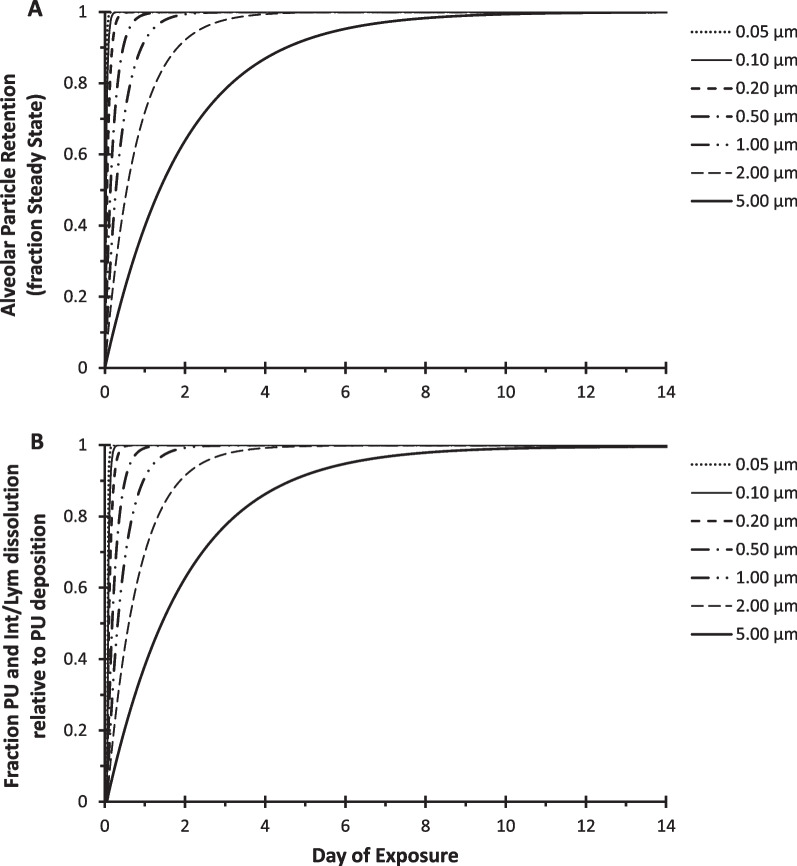
Effect of initial particle size (highly soluble, dissolution rate of 1 × 10^−4^ g/cm^2^ per day; density of 3 g/cm^3^) on time to steady state in the alveolar region (Panel **A**) and dissolution from alveolar and interstitial/lymph compartments (Panel **B**). Above results are for 1 µg deposition per day in the alveolar region and are invariant to/with a person’s age or activity level

**Fig. 5:**
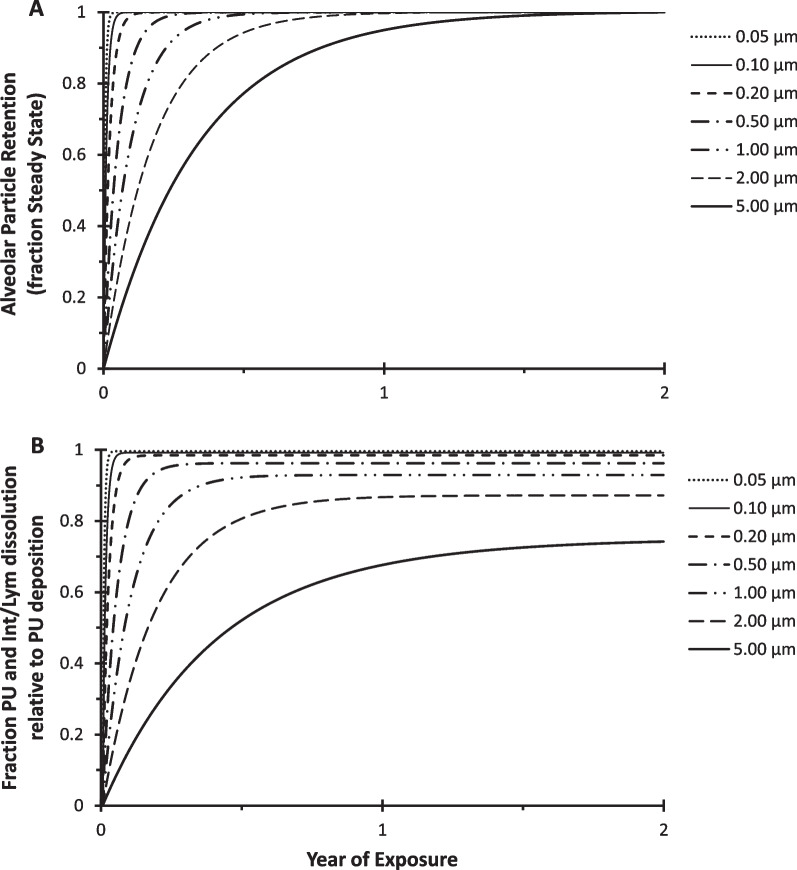
Effect of initial particle size (moderately soluble, dissolution rate of 1 × 10^−6^ g/cm^2^ per day; density of 3 g/cm^3^) on time to steady state in the alveolar region (Panel **A**) and dissolution from alveolar and interstitial/lymph compartments (Panel **B**). Above results are for 1 µg deposition per day in the alveolar region and are invariant to/with a person’s age or activity level

**Fig. 6:**
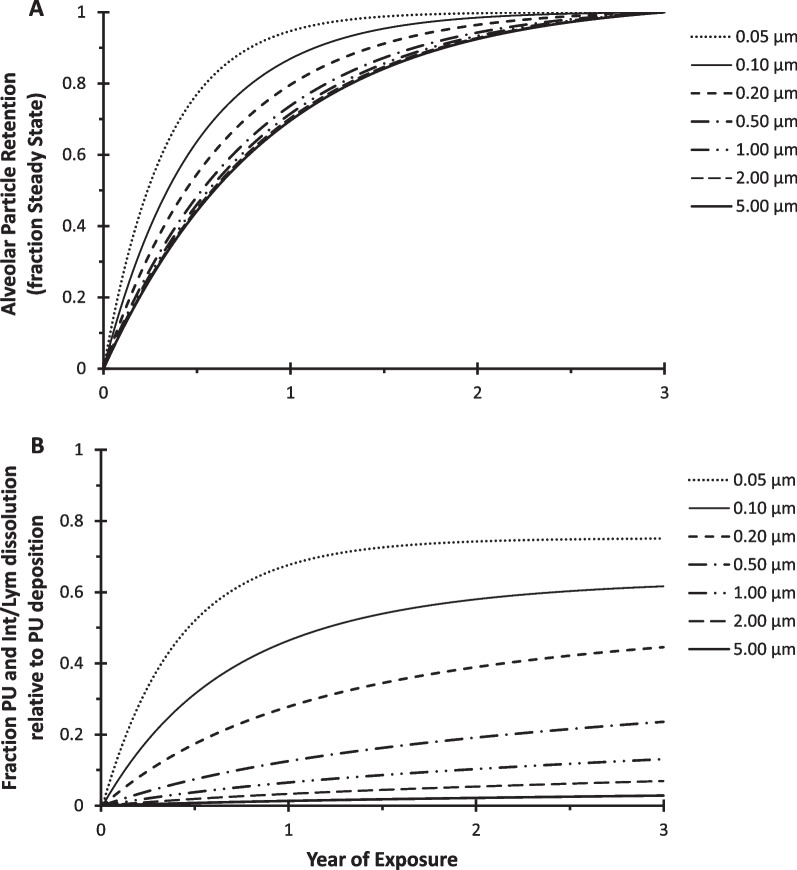
Effect of initial particle size (poorly soluble, dissolution rate of 1 × 10^−8^ g/cm^2^ per day; density of 3 g/cm^3^) on time to steady state in the alveolar region (Panel **A**) and dissolution from alveolar and interstitial/lymph compartments (Panel **B**). Above results are for 1 µg deposition per day in the alveolar region and are invariant to/with a person’s age or activity level

Figures 4, 5 and 6 illustrate the time required for monodisperse aerosols of differing solubilities (highly, moderately, and poorly) depositing in the alveolar region to reach a balance between the rate of particle accumulation and removal by clearance (i.e., to TB and Int/Lym compartments) and dissolution from the PU and Int/Lym compartments normalized to the amount of deposition in the PU region. These figures were calculated using the compartmental model illustrated in Fig. 1 along with the equations for calculating regional particle burden and dissolution (Eqs. 13–17). Not surprisingly, these figures show that the time to reach steady state increases with decreasing dissolution rate and increasing particle size. For highly soluble particles, Fig. 4 shows that steady state burden in the PU region is reached within about a week and that by 2 weeks there is a near constant rate of particle dissolution from the PU and Int/Lym compartments that is equal to the deposition within the PU region. For moderately soluble particles, Fig. 5 shows that steady state PU retention and dissolution from the PU and Int/Lym compartments are generally reached within about a 2-year period. However, in contrast to the highly soluble particles in Figs. 4 and 5 shows that moderately soluble particles larger than ~ 0.2 µm reach a steady state in which the rate of dissolution that is less than the rate of deposition in the PU region. This is due to their clearance from the PU to the TB region. For poorly soluble particles, Fig. 6 shows that still longer periods of time are required to reach steady state burden in the PU region and that clearance from the PU region reduces the amount of material available for dissolution even for the smallest particle sizes considered.

**Fig. 7:**
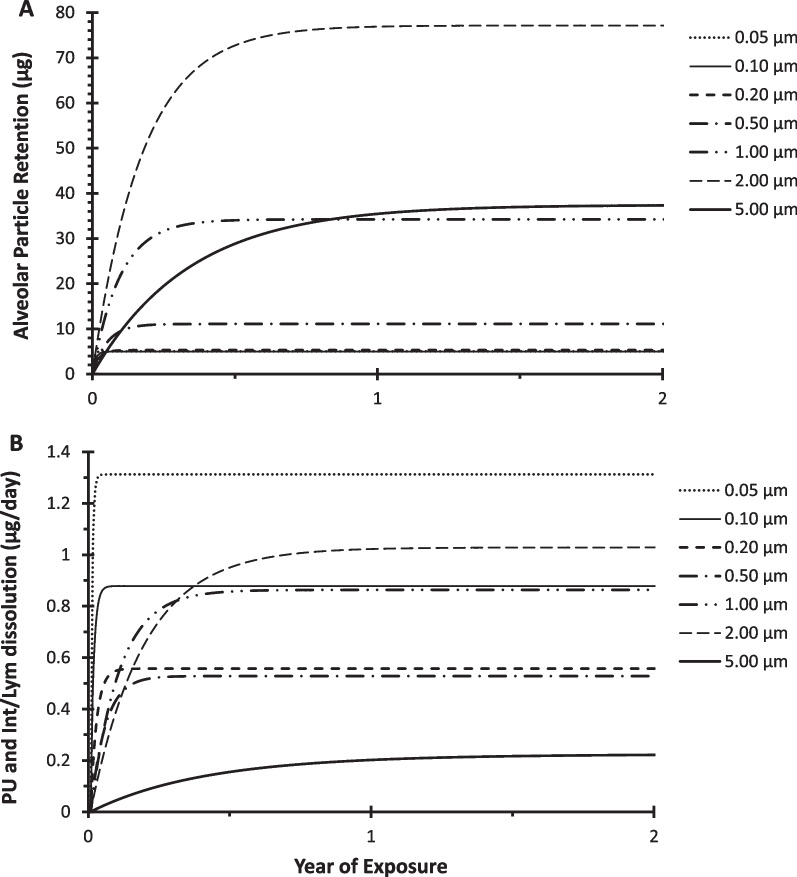
Effect of inhaled particle size (moderately soluble, dissolution rate of 1 × 10^−6^ g/cm^2^ per day; density of 3 g/cm^3^) on alveolar burden (Panel **A**) and daily dissolution from pulmonary and interstitial/lymph compartments (Panel **B**). Monodisperse aerosol (concentration = 1.0 µg/m^3^) of indicated sized particles were inhaled for 8-h per day by an adult male exposed while sitting at rest

Figures 7 illustrates the effect of inhaled particle size on pulmonary burden and dissolution from pulmonary and interstitial/lymphatics. The mass deposition rate was calculated for moderately soluble particles using Eq. 10. The exposure simulation is for an adult male exposed while sitting at rest for an 8-h period to a monodisperse aerosols having a Pb concentration of 1.0 µg/m^3^. The overall inhaled volume of air was constant at 4.3 m^3^/day (i.e., the 8-h exposure period) for the activity of sitting. In Fig. 7A, the highest pulmonary burdens are predicted for 2 µm particles due to the combination of a high deposition fraction of 0.27 in the PU region and slow dissolution (0.013 day^−1^; t_1/2_, 55 days). This may be contrasted with the low burden of 0.05 µm particles due to small particle size causing a high dissolution rate (0.51 day^−1^; t_1/2_, 1.4 days) despite a high deposition fraction of 0.31 in the PU region. In Fig. 7B, the dissolution of 0.05 µm particles is nearly the same as for highly soluble particles (not illustrated) due the large surface area per unit mass for dissolution. Although the deposition fraction in the PU region of only 0.069 for 5 µm particles was the lowest relative to other sizes illustrated, it reached the second highest pulmonary retention due to slow dissolution (0.005 day^−1^; t_1/2_, 137 days).

**Fig. 8:**
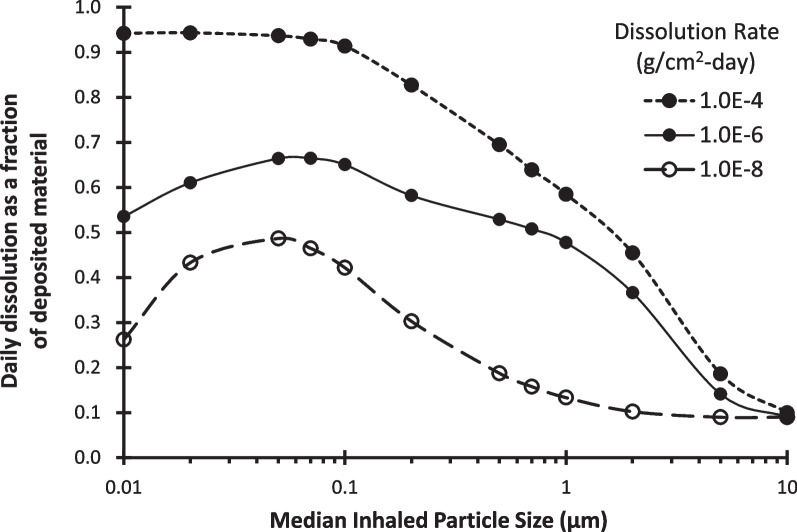
Total dissolution (all lung regions and the gastrointestinal tract) of particles normalized to deposition of material in the respiratory tract. Monodisperse aerosol (concentration = 1.0 µg/m^3^) of indicated sized particles (density = 3 g/cm^3^) were inhaled for 8-h/day for 3 years by adult male exposed while sitting at rest. The daily dissolution reflects that occurring on day 1095 of exposure with gastrointestinal absorption of 10%

**Fig. 9:**
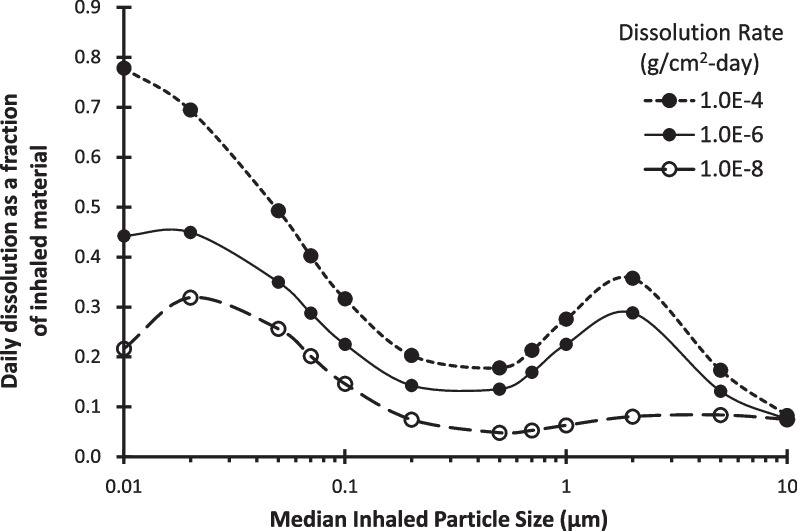
Total dissolution (includes all lung regions and the gastrointestinal tract) of particles normalized to amount of material inhaled. Monodisperse aerosol (concentration = 1.0 µg/m^3^) of indicated sized particles (density = 3 g/cm^3^) were inhaled for 8-h/day for 3 years by adult male exposed while sitting at rest. The daily dissolution reflects that occurring on day 1095 of exposure with gastrointestinal absorption of 10%

Figure 8 illustrates daily total particle dissolution normalized by daily deposited dose in the respiratory tract and daily inhaled dose. On this figure, dissolution equals the absorption of material into the blood assuming 100% of material dissolved (i.e., after dissolving, the material does not become bound and unavailable for absorption into blood) in the lungs and 10% absorption of material cleared to the gastrointestinal tract. Approximately, 95% of all material associated with highly soluble ultrafine (i.e., ≤ 0.1 µm) particles deposited within the respiratory tract is expected to be dissolved and absorbed into the blood. This fraction then gradually decreases to only 10% of 10 µm particles since their absorption effectively occurs only within the gastrointestinal tract. Moderate and poorly soluble particles show lower amounts of absorption. Figure 9 illustrates daily total particle dissolution and absorption into blood normalized by daily inhaled dose. For both highly and moderately soluble ultrafine particles (i.e., ≤ 0.1 µm), greater than 30% of inhaled material is expected to be absorbed into the blood. There is also a peak in the absorption for highly and moderately soluble particles at 2 µm with decreased absorption above and below this size. For poorly soluble particles, absorption is expected to be < 10% for all sized particles, except ultrafines.

All of the results presented in figures and Table 2 were for monodisperse aerosols. For polydisperse aerosols (GSD > 1.15), it is important to recognize that the median particle size depositing in each region of the respiratory tract will differ from the inhaled median diameter. In general, the median particle size predicted to deposit in lung regions decreases with distal progression. The pattern may be reversed for submicron particles where the median sized depositing in the PU region is sometimes larger than that depositing in the TB region. This is important because, as illustrated in figures and Table 2, dissolution rates are affected by the size of the deposited particles. Tables 3 and 4 for males and females, respectively, provide the estimated mass median diameter of particles predicted to deposit in each respiratory tract region for inhaled polydisperse (GSD = 2.0) aerosols of several mass median particle sizes (namely, 0.1, 0.5, 1, 5, and 10 µm) and human activity levels (sitting, light and heavy exercise). The predicted results for males and females are quite similar with females tending to have slightly smaller median particle diameters depositing in regions than males. Table 5 provides deposition fractions and daily dose rates for males and females under the same conditions as for Tables 3 and 4. With the exception of the deposition fractions in the ET regions in for females sitting at rest, the deposition fractions in females were always slightly less than in males. However, in all cases, the daily deposited dose rate (µg of the compound of interest depositing per day in respiratory tract regions) was greater in males than females. For the single case where daily dose rates appear to be the same in Table 5, 0.02 µg/day for 0.1 µm particles depositing in the ET region during the activity of sitting, the male and female dose rates with an additional significant figure were 0.023 and 0.020 µg/day, respectively.

**Table 3:** Median particle diameter depositing in regions of an adult male respiratory tract following inhalation of polydisperse aerosols (GSD = 2.0)

Activity	Region	Inhaled mass median diameter (µm)^a^				
		0.1	0.5	1	5	10
		Deposited mass median diameter (5th, 95th percentiles)				
Sitting	ET	0.099 (0.025, 0.332)	0.869 (0.232, 2.24)	1.61 (0.56, 4.10)	5.55 (2.05, 14.1)	9.05 (3.43, 23.0)
	TB	0.074 (0.024, 0.241)	0.520 (0.142, 1.78)	1.25 (0.35, 3.63)	4.56 (1.77, 10.2)	6.57 (2.80, 14.5)
	PU	0.078 (0.028, 0.248)	0.611 (0.153, 1.74)	1.18 (0.38, 2.77)	2.77 (1.24, 5.43)	3.61 (1.71, 6.49)
Light exercise	ET	0.111 (0.027, 0.371)	0.866 (0.251, 2.197)	1.56 (0.55, 4.10)	5.79 (2.07, 14.9)	9.55 (3.59, 25.1)
	TB	0.076 (0.024, 0.248)	0.498 (0.142, 1.74)	1.23 (0.34, 3.89)	4.70 (1.89, 9.50)	6.17 (2.93, 13.2)
	PU	0.073 (0.026, 0.236)	0.612 (0.149, 1.85)	1.26 (0.39, 2.95)	2.84 (1.33, 5.02)	3.47 (1.76, 5.72)
Heavy exercise	ET	0.121 (0.028, 0.405)	0.853 (0.266, 2.16)	1.51 (0.54, 4.05)	5.74 (2.04, 14.9)	9.54 (3.58, 25.5)
	TB	0.079 (0.025, 0.258)	0.514 (0.148, 1.89)	1.36 (0.35, 3.95)	4.19 (1.94, 8.42)	5.34 (2.73, 11.9)
	PU	0.071 (0.024, 0.229)	0.592 (0.144, 1.82)	1.24 (0.38, 2.72)	2.49 (1.24, 4.07)	2.89 (1.68^b^, 4.48)

*ET* extrathoracic, *TB* tracheobronchial, *PU* pulmonary

^a^Deposition calculations are for a particle density of 3 g/cm^3^ in this example

^b^The first particle bin contains 7.0% of the mass deposited in this region

**Table 4:** Median particle diameter depositing in regions of an adult female respiratory tract following inhalation of polydisperse aerosols (GSD = 2.0)

Activity	Region	Inhaled mass median diameter (µm)^a^				
		0.1	0.5	1	5	10
		Deposited mass median diameter (5th, 95th percentiles)				
Sitting	ET	0.092 (0.025, 0.321)	0.875 (0.229, 2.25)	1.62 (0.56, 4.09)	5.39 (2.03, 13.5)	8.65 (3.33, 21.4)
	TB	0.073 (0.024, 0.239)	0.525 (0.142, 1.78)	1.24 (0.35, 3.47)	4.34 (1.68, 10.2)	6.49 (2.67, 14.9)
	PU	0.078 (0.028, 0.246)	0.608 (0.151, 1.74)	1.18 (0.38, 2.71)	2.66 (1.22, 5.19)	3.44 (1.68^b^, 6.24)
Light exercise	ET	0.109 (0.027, 0.364)	0.869 (0.247, 2.20)	1.57 (0.56, 4.10)	5.77 (2.07, 14.8)	9.51 (3.58, 24.9)
	TB	0.076 (0.024, 0.247)	0.498 (0.142, 1.74)	1.24 (0.34, 3.92)	4.73 (1.90, 9.56)	6.21 (2.95, 13.2)
	PU	0.074 (0.026, 0.236)	0.607 (0.148, 1.84)	1.25 (0.39, 2.94)	2.83 (1.32, 5.02)	3.47 (1.75, 5.73)
Heavy exercise	ET	0.119 (0.025, 0.399)	0.859 (0.264, 2.17)	1.52 (0.55, 4.06)	5.73 (2.04, 14.9)	9.52 (3.58, 25.4)
	TB	0.079 (0.025, 0.258)	0.522 (0.149, 1.96)	1.43 (0.36, 3.94)	4.09 (1.94, 8.28)	5.23 (2.68, 11.7)
	PU	0.071 (0.024, 0.227)	0.580 (0.142, 1.81)	1.23 (0.37, 2.67)	2.43 (1.22, 3.91)	2.79 (1.68^c^, 4.28)

^a^Deposition calculations are for a particle density of 3 g/cm^3^ in this example

^b^The first particle bin contains 5.3% of the mass deposited in this region

^c^The first particle bin contains 7.8% of the mass deposited in this region

**Table 5:** Particle deposition fraction and dose rates in regions of adult respiratory tract following inhalation of polydisperse aerosols (GSD = 2.0)

Activity	Region	Sex	Inhaled mass median diameter (µm)^a^				
			0.1	0.5	1	5	10
			Deposition fraction (Dose rate, µg/day)^b^				
Sitting	ET	Male	0.04 (0.02)	0.11 (0.06)	0.22 (0.12)	0.62 (0.33)	0.62 (0.33)
		Female	0.05 (0.02)	0.12 (0.04)	0.25 (0.10)	0.65 (0.25)	0.62 (0.24)
	TB	Male	0.12 (0.07)	0.07 (0.04)	0.09 (0.05)	0.12 (0.07)	0.08 (0.05)
		Female	0.13 (0.05)	0.07 (0.03)	0.09 (0.03)	0.11 (0.04)	0.07 (0.03)
	PU	Male	0.21 (0.11)	0.16 (0.08)	0.20 (0.11)	0.10 (0.05)	0.03 (0.02)
		Female	0.17 (0.07)	0.12 (0.05)	0.15 (0.06)	0.07 (0.03)	0.02 (0.01)
Light exercise	ET	Male	0.03 (0.05)	0.11 (0.16)	0.22 (0.33)	0.62 (0.92)	0.67 (0.99)
		Female	0.03 (0.04)	0.10 (0.13)	0.22 (0.27)	0.61 (0.76)	0.66 (0.82)
	TB	Male	0.10 (0.15)	0.06 (0.09)	0.07 (0.10)	0.11 (0.17)	0.08 (0.11)
		Female	0.10 (0.13)	0.06 (0.07)	0.07 (0.09)	0.12 (0.15)	0.08 (0.10)
	PU	Male	0.21 (0.31)	0.14 (0.20)	0.18 (0.27)	0.11 (0.16)	0.03 (0.05)
		Female	0.22 (0.27)	0.14 (0.17)	0.18 (0.23)	0.11 (0.14)	0.03 (0.04)
Heavy exercise	ET	Male	0.03 (0.09)	0.12 (0.36)	0.25 (0.73)	0.65 (2.0)	0.70 (2.1)
		Female	0.03 (0.08)	0.12 (0.32)	0.24 (0.66)	0.65 (1.8)	0.70 (1.9)
	TB	Male	0.09 (0.27)	0.06 (0.18)	0.08 (0.23)	0.13 (0.39)	0.07 (0.22)
		Female	0.09 (0.24)	0.06 (0.16)	0.08 (0.22)	0.14 (0.37)	0.08 (0.21)
	PU	Male	0.20 (0.58)	0.11 (0.34)	0.14 (0.42)	0.07 (0.20)	0.02 (0.05)
		Female	0.19 (0.50)	0.10 (0.28)	0.13 (0.34)	0.06 (0.15)	0.01 (0.03)

^a^Deposition calculations are for a particle density of 3 g/cm^3^ in this example

^b^Dose rates are for an inhaled aerosol concentration containing 1 µg of the compound of interest per cubic meter

## Discussion

Predicting pharmacokinetics of inhaled particulate aerosols requires accurate simulation of the processes that govern absorption of inhaled particles. Within the respiratory tract, the key processes that must be represented in models include regional deposition of inhaled particles, mucociliary clearance to the gastrointestinal tract and dissolution within the respiratory tract. A computational challenge in simulating dissolution of a particles deposited in the respiratory tract is that the rate of loss of particle mass from the dissolving particle is a function of particle size, which changes as dissolution proceeds and reshapes the particle (i.e., surface area/mass ratio). While this nonlinear process can be simulated by numerically integrating the particle dissolution equations described in the Methods, models commonly used to simulate the fate of inhaled particles (e.g., ICRP) simplify particle dissolution by assuming constant rates. Herein, we offer an alternative approach that uses first order rate coefficients to approximate the time-dependent loss of particle mass from dissolution of a size-evolving particle. This approach can be easily implemented in a spreadsheet. Using this approach, in concert with particle deposition and clearance models, we illustrate the importance of size-dependent dissolution in predicting absorption of inhaled particulate aerosols. Our simulations showed that the balance between accumulation in the pulmonary region and removal by normal clearance and particle dissolution depends on both the solubility and size of the deposited particle (Figures 4, 5, 6 and 7). We showed (Fig. 6 and Table 2) that it can take years to decades for poorly soluble particles to reach steady state alveolar retention when a balance is achieved between rates of particle deposition, normal particle clearance, and dissolution. The rates of particle dissolution are directly proportional to particle size depositing in regions of the respiratory tract. These results suggest that simple classifications of particles into dissolution rates, independent of particle size, such as ICRP characterizations of F, M, and S type dissolution rates can result in substantial errors in the prediction of dissolution and absorption of inhaled particulate aerosols, especially for particles ≤ 1 µm in diameter. A two-order of magnitude difference in particle size is equivalent to a two-order of magnitude difference in dissolution rates. That is, in theory, the first-order dissolution rate of highly soluble 10 µm particles is the same as the first-order dissolution rate of moderately soluble 0.1 µm particles (Table 2). We showed that, for particle sizes 0.01–2 µm, assuming rapid dissolution of less soluble particles would overestimate dissolution and absorption compounds associated with the deposited particles which actually dissolve more slowly (Fig. 8). Absorption from large particles (i.e., 10 µm) is less sensitive to solubility, because these large particles deposit predominately in the extrathoracic region and are quickly cleared to the stomach.

We now discuss the implications of our findings in relation to various forms of Mn for which extensive studies of blood Mn and PBPK modeling are available. Reference [17] recommends the default Type M for Mn in the absence of specific information on which the exposure material can be assigned to an absorption type (e.g., if the form is unknown, or if the form is known but there is no information available on the absorption of that form from the respiratory tract). Data on particle density and dissolution rates are available from animal studies of Mn exposure and Mn PBPK modeling. Table 6 provides information on Mn oxides, Mn sulfate, and Mn chloride densities from Ref. [18] and Mn phosphate (hureaulite) density from mineralogical websites [19, 20]. In vitro dissolution rates are very dependent on solution type [21]. The influence of particle solubility on the delivery of inhaled Mn to the rat brain has been studied [22]. The authors exposed rats by inhalation for 14-day to aerosols of Mn sulfate (MnSO_4_) and Mn tetroxide (Mn_3_O_4_). The authors reported that the solubility of MnSO_4_ is 3600-times that of the Mn_3_O_4_. Animals exposed to MnSO_4_ (3 mg Mn/m^3^) had lower lung and higher olfactory bulb and striatal Mn concentrations compared with levels achieved following similar Mn_3_O_4_ exposures. Ref. [24] provides a PBPK model for Mn in monkeys and included a comparison with experimental data. The PBPK model assumed rapid dissolution of particles regardless of their form (i.e., soluble Mn sulfates versus poorly soluble Mn oxides) once deposited within the respiratory tract. However, the authors found it necessary to apply deposition adjustment factors (Table 3 of their paper) to reduce Mn available in the lungs for dissolution of poorly soluble Mn oxide particles. The authors of Ref. [24] recognized the need for these deposition adjustment factors “… to account for different particle size fractions and reduced bioavailability of less soluble Mn forms in lung tissues.” These deposition adjustment factors were derived to match the PBPK model predictions with to globus pallidus Mn concentrations observed in three experimental studies of monkeys exposed to Mn oxides (MnO_2_ or Mn_3_O_4_) by inhalation. The deposition adjustment factors were 0.0028 (MnO_2_), 0.03 (MnO_2_), and 0.4 (Mn_3_O_4_). Reciprocals of these factors (representing the magnitude of overestimation due to assuming MnSO_4_ solubility) show that assuming in their PBPK model that poorly soluble Mn oxides reached the blood at a rate similar to highly soluble Mn sulfate resulted in overestimates of Mn reaching the brain by 2.5-, 33-, and 360-times. The deposition adjustment factors included in Ref. [24] demonstrate the problem with the conservative approach in PBPK models of assuming the complete and instantaneous dissolution metals from poorly soluble particles. Unfortunately, those deposition adjustment factors did not have a mechanistic basis that was generalizable (or scalable) to other scenarios, such as other inhaled particle sizes, density, solubility, or exposure durations.

**Table 6:** Particle density and dissolution rates for several Mn forms

Form	Density (g/cm^3^)	Dissolution rate (g/cm^2^ per day)	1 µm dissolution (fraction per unit time)	Dissolution rate notes
MnSO_4_	3.25	1.1 × 10^−4^ to 7.5 × 10^−4^	0.6–0.1 per hr	Hatch and Gamble solutions [21]
MnCl_2_	2.98	2.9 × 10^−5^	0.6 per day	Estimated [20]
MnHPO_4_	3.18	1.4 × 10^−5^ to 2.1 × 10^−6^	0.3–0.04 per day	Hatch and Gamble solutions [21]
MnHPO_4_	3.18	1.1 × 10^−5^	0.2 per day	Estimated [23]
Mn_3_O_4_	4.84	2.1 × 10^−7^ to 5.3 × 10^−8^	0.003–0.001 per day	Hatch and Gamble solutions [21]
MnO_2_	5.08	5.9 × 10^−8a^	0.001 per day^a^	Estimated [6]

^a^Ref. [6] provides lung clearance data for Mn^54^O_2_ and uranium oxide (U^235^O_2_) in dogs. The MnO_2_ particles (count median diameter, 0.07 µm; GSD, 1.66) and UO_2_ particles (count median diameter, 0.33 µm; GSD, 1.80) had biological clearance t_1/2_ from the lungs of 38 and 190 days, respectively. Adjusted t_1/2_ for the respective median particle diameters reveal the elimination rates to be quite similar between the MnO_2_ (0.33/0.07 × 38 days = 180 days vs. 190 days for UO_2_) and UO_2_ (0.07/0.33 × 190 days = 40 days vs. 38 days for MnO_2_). Density was not included in calculations since clearance measurements normalize to mass deposited. Assuming the faster clearance rate of about 1% per day of MnO_2_ relative to UO_2_ is due to dissolution, for 0.07 µm MnO_2_ particles corresponds to dissolution rate of 5.9 × 10^−8^ g/cm^2^ per day

**Fig. 10:**
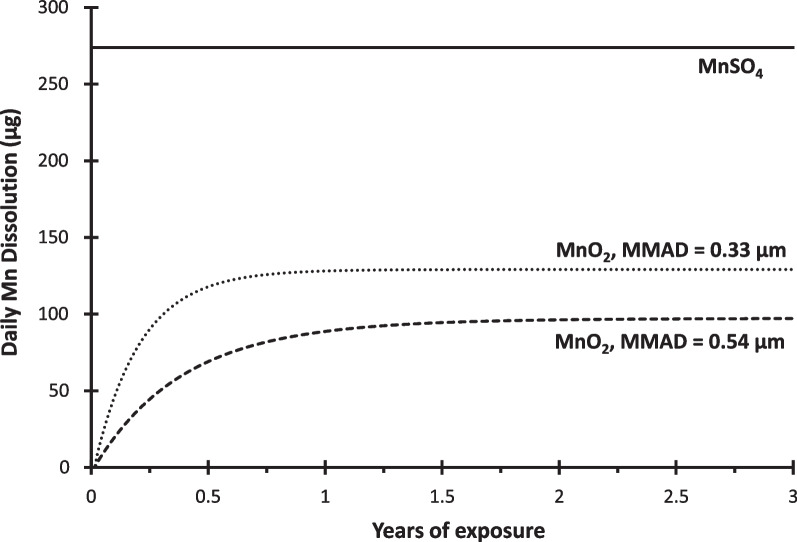
Total dissolution (includes all lung regions and the gastrointestinal tract) available for absorption into blood. Mn-laden aerosol (concentration = 100 µg-Mn/m^3^) of indicated sized particles (density = 5.08 g/cm^3^) were inhaled while a light exercise level of activity for 8-h/day, 5 days/week, for 3 years by adult male exposed by welding. The 5 days/week exposure was implemented by reducing the daily amount of air inhaled by 5/7 to smooth the illustrated lines. The Mn form from welding was assumed to be MnO_2_, the MnSO_4_ line illustrates the daily dissolution rate of a highly soluble Mn form. The daily dissolution includes gastrointestinal absorption of 5% for MnSO_4_ and 0.05% for MnO_2_

**Fig. 11:**
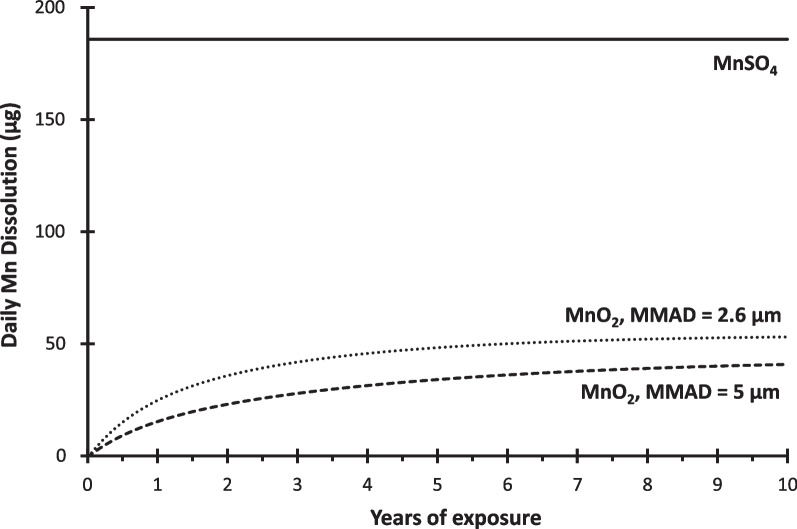
Total dissolution (includes all lung regions and the gastrointestinal tract) available for absorption into blood. Mn-laden aerosol (concentration = 100 µg-Mn/m^3^) of indicated sized particles (density = 5.08 g/cm^3^) were inhaled while a light exercise level of activity for 8-h/day, 5 day/week, for 3 years by adult male exposed by during ferroalloy work (MMAD = 2.6 µm) and battery manufacturing (MMAD = 5 µm). The 5 days/week exposure was implemented by reducing the daily amount of air inhaled by 5/7 to smooth the illustrated lines. The Mn form that workers were exposed to was assumed to be MnO_2_, the MnSO_4_ line illustrates the daily dissolution rate of a highly soluble Mn form. The daily dissolution includes gastrointestinal absorption of 5% for MnSO_4_ and 0.05% for MnO_2_

A Mn PBPK model was used to estimate blood Mn concentrations of workers exposed to Mn [2]. Aerosol size distributions were expressed as lognormal about their mass median aerodynamic diameter (MMAD). The occupations and Mn aerosols size distributions were welders (MMAD = 0.33 µm, GSD = 4.0; and MMAD = 0.54 µm, GSD = 2.4), ferroalloy smelter workers (MMAD = 2.6 µm, GSD = 4.5), and Mn oxide production workers (MMAD = 5.0 µm, GSD = 3). As a conservative approach, the high solubility of MnSO_4_ was used rather than the very low solubility of a Mn oxide [2]. In Figs. 10 and 11, we show the implications of assuming the high solubility of MnSO_4_ versus poorly solubility of MnO_2_. For our simulations, we used dissolution rates of 4.3 × 10^−4^ for MnSO_4_ and 5.9 × 10^−8^ for MnO_2_. Additionally, the average DF_r_ (i.e., regional deposition fraction) of the two MnO_2_ aerosols on each figure were used as the DF_r_ of MnSO_4_. For welding sized particles (Fig. 10), our model predicted that it would take nearly a year before a steady state rate of daily Mn dissolution/absorption is reached. At 3 years, well after a steady state has been reached, the 0.54 and 0.33 µm MMAD MnO_2_ aerosols were predicted to have daily dissolution rates that were 35 and 47% of those estimated for MnSO_4_. At 6 months of exposure, the cumulative dissolution of the 0.54 and 0.33 µm MMAD aerosols were predicted to be 15 and 29% of those estimated for MnSO_4_. For ferroalloy smelter and Mn oxide production sized particles (Fig. 11), a steady state rate is not achieved by 10 years of MnO_2_ exposure. After 10 years of exposure to the 5.0 and 2.6 µm MMAD MnO_2_ aerosols, the daily dissolution rates were predicted to be 22 and 29% of those estimated for MnSO_4_. However, at 10 years of exposure, the cumulative dissolution of the 5.0 and 2.6 µm MMAD MnO_2_ aerosols were predicted to be only 17 and 23% of those estimated for MnSO_4_. Considering a shorter exposure duration of 0.5 year, the cumulative dissolution of the 5.0 and 2.6 µm MMAD MnO_2_ aerosols were predicted to be only 3 and 4%, respectively, of those estimated for MnSO_4_. Conceptually, the percentages of Mn oxide dissolution relative to Mn sulfates discussed here are similar, in concept, to deposition adjustment factors in Ref. [24] that were needed to account for the reduced bioavailability of less soluble Mn oxides relative to Mn sulfates.

**Fig. 12:**
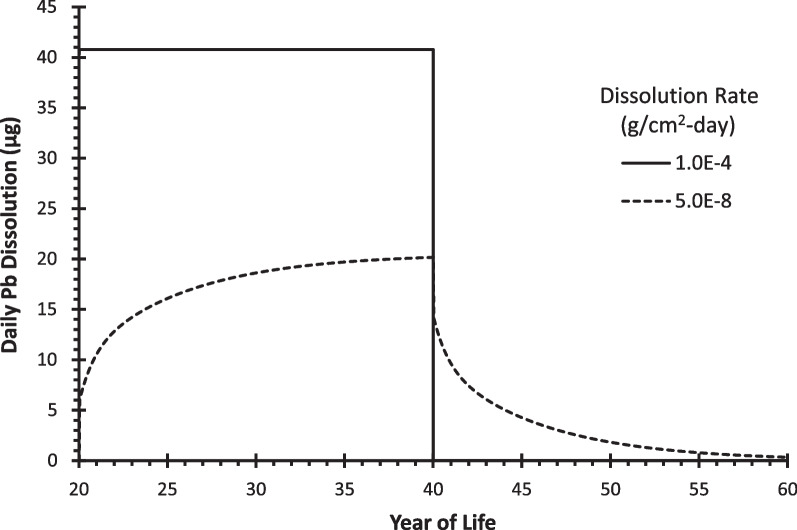
Total dissolution (includes all lung regions and the gastrointestinal tract) available for absorption into blood. Lead tetraoxide aerosol (concentration = 45 µg-Pb/m^3^) of monodisperse 3.0 µm MMAD particles (density = 8.3 g/cm^3^) were inhaled by an adult male sitting at rest for 8-h/day, 5 day/week, from age 20 to 40 years. The 5 days/week exposure was implemented by reducing the daily amount of air inhaled by 5/7 to smooth the illustrated lines. The daily dissolution includes gastrointestinal absorption of 9%

In Fig. 12, we modeled a hypothetical scenario of a worker exposed for 20 years to red lead oxide [i.e., lead tetraoxide, Pb_3_O_4_] at a concentration of 50 µg-Pb_3_O_4_/m^3^, the threshold limit value established by the American Conference of Governmental Industrial Hygienists. After 20 years of exposure, the daily Pb dissolution rate of poorly soluble particles was predicted to be 49% of highly soluble particles. However, notice that although the dissolution of highly soluble particles was predicted to cease within a day, the dissolution of poorly soluble particles continues due to particle burden in the pulmonary region and interstitial/lymphatics. At 60 years of age, 20 years after exposure ceased, the cumulative dissolution of poorly soluble particles was predicted to be 50% of that for highly soluble particles at 40 years of age. Thus, the lungs would continue to be a source of Pb for absorption into the blood many years after exposure ceased.

## Conclusion

We found that assuming high solubility of poor soluble materials will result in an overestimate of the daily rate of particle dissolution. Although assuming high solubility is a conservative approach to estimate maximal rates of particle dissolution in the respiratory tract for absorption into the blood, this approach will always underestimate pulmonary particle burden and overestimate the movement of materials from the lungs and distribution in extrapulmonary tissues. The implication to PBPK modeling (e.g., with consideration to Fig. 11) is that, if PBPK modeled blood concentrations match those observed in exposure workers after only 6 months of exposure, then other factors in the PBPK model are offsetting the roughly 20–40-times greater cumulative dissolution of MnSO_4_ relative to MnO_2_. Over a longer 10-year exposure period, PBPK models could still be offsetting the roughly 4–6-times greater cumulative dissolution of MnSO_4_ relative to MnO_2_. Additionally, the PBPK models are underestimating pulmonary and interstitial/lymphatic particle burden that can lead to additional material dissolution and absorption into the blood years after exposure has ceased. For poorly soluble Pb-laden particles becoming deposited in the pulmonary region of workers, the lungs could act as a storage compartment in addition to cortical bone that can contribute to blood Pb for years after exposure ceases. In addition to modeling dose rates for particle deposition into the lung, PBPK modeling of pulmonary and extrapulmonary tissues of moderately and poorly soluble materials can be improved by including estimates of lung burden and dissolution over time.

## List of symbols

∆t: Time-step
*k*: Particle dissolution rate (g/cm^2^ particle surface area per day)
λ_Int_: First-order rate constant for particle transfer from pulmonary region to interstitial space
λ_r_: First-order particle dissolution rate in a region (ET, TB, or PU) of the respiratory tract
λ_r__,Clr_: First-order rate constant for particle clearance from a region (ET, TB, or PU) of the respiratory tract
*ρ*: Particle density (g/cm^3^)
σ_g_: Geometric standard deviation (also GSD)
B_Int_: Interstitial particle burden
B_r_: Particle burden in a region (ET, TB, or PU) of the respiratory tract
Cd_i_: Airborne concentration (µg/m^3^) of a compound associated a given particle size interval
d_i_: Particle size associated with a percentile of a particle size distribution
d_r_(t): The particle size in region of the respiratory tract at time t
d_r,initial_: Initial median physical particle size deposited in a region of the respiratory tract
DF_r,di_: Deposition fraction given particle size within a region of the respiratory tract
Dissol_r_: Particle dissolution from respiratory tract regions (ET, TB, PU, or Int)
dissolution(t): Instantaneous dissolution rate (g/day) at time t
ET: Extrathoracic region of respiratory tract
*f*: Breathing frequency
F: Characterized as fast rate of dissolution
*f*_*r*_ (i): Estimated the fraction of a particle dissolved per day
*f*_dr,initial_: Fraction dissolved per day of particle size initially depositing in a region of the respiratory tract
GI: Gastrointestinal
GSD: Geometric standard deviation (also σ_g_)
ICRP: International Commission on Radiological Protection
Int: Interstitial region of respiratory tract
m_*frac*_: Fraction of particle mass remaining
m(t): Remaining particle mass at time t
M: Characterized as moderate rate of dissolution
$\dot{M}_{r}$: Overall mass deposition rate (µg/day) in a region of the respiratory tract
$\dot{M}_{{r,d_{i} }}$: Mass deposition rate (µg/day) of specific particle size in a region of the respiratory tract
Mn: Manganese
MnO_2_: Mn oxide
Mn_3_O_4_: Mn tetroxide
MnSO_4_: Mn sulfate
MMAD: Mass median aerodynamic diameter
MPPD: Multiple-path particle dosimetry
MSS: Sum of squares
P: Percentile of cumulative probability distribution
Pb: Lead
PBPK: Physiologically based pharmacokinetic
PU: Pulmonary (alveolar) region of respiratory tract
*r*: A region of the respiratory tract
*r*^2^: Goodness of fit between model and data
RSS: Residual sum of squares
S: Characterized as slow rate of dissolution
Stomach_ET_: Clearance particles from the ET region to the stomach
TB: Tracheobronchial region of respiratory tract
t: Time
t_*frac*_: Cumulative time for dissolution to fraction of particle mass remaining
TSS: Total corrected sum of squares
V_T_: Tidal volume
z(P): Number of standard deviations from the mean for the normal distribution at a given cumulative percentile or probability
